# Exercise-Induced Release of Cardiac Troponins in Adolescent vs. Adult Swimmers

**DOI:** 10.3390/ijerph18031285

**Published:** 2021-02-01

**Authors:** Rafel Cirer-Sastre, Francisco Corbi, Isaac López-Laval, Luis Enrique Carranza-García, Joaquín Reverter-Masià

**Affiliations:** 1 Institut Nacional d’Educació Física de Catalunya (INEFC), Universitat de Lleida (UdL), 25192 Lleida, Spain; fcorbi@inefc.es; 2 Facultad de Ciencias de la Salud y del Deporte, Universidad de Zaragoza, 50009 Zaragoza, Spain; isaac@unizar.es; 3 Facultad de Organización Deportiva (FOD), Universidad Autónoma de Nuevo León (UANL), San Nicolás de los Garza 66455, Mexico; luis.carranzagr@uanl.edu.mx; 4 Departament de Didàctiques Específiques, Universitat de Lleida (UdL), 25003 Lleida, Spain; joaquim.reverter@udl.cat

**Keywords:** biomarkers, heart damage, swimming, growth

## Abstract

To examine the exercise-induced release of cardiac troponin T (cTnT) in adolescent and adult swimmers. Thirty-two trained male (18 adolescents, 14 adults) swam at maximal pace in a 45 min distance trial, and blood samples were drawn before, immediately and 3 h after exercise for subsequent cTnT analysis and comparison. Having comparable training experience and baseline values of cTnT (*p* = 0.78 and *p* = 0.13), adults exercised at lower absolute and relative intensity (*p* < 0.001 and *p* < 0.001, respectively), but presented higher immediate cTnT after exercise than adolescents (*p* < 0.001). Despite that, peak concentrations were observed at 3 h post exercise and peak elevations were comparable between groups (*p* = 0.074). Fourteen (44%) apparently healthy subjects exceeded the cutoff value for myocardial infarction (MI). Adolescents presented a delayed elevation of cTnT compared with adults, but achieved similar peak values.

## 1. Introduction

Elevations of serum cardiac troponin (cTn) are the preferred criteria to diagnose myocardial injury [1]. Concretely, the release of cTn into the bloodstream has been related to different clinical scenarios and explained by mechanisms of release such as myocardial ischemia, inflammatory and immunological processes, trauma, drugs or toxins [1,2]. It is of particular interest though that exercise frequently evokes elevations of serum cardiac troponin (cTn), that peak approximately 3 h after exercise and return to basal concentrations within the subsequent 24 h [3]. Furthermore, a growing body of evidence suggests that cTn elevations induced by exercise occur in apparently healthy athletes and might respond to a physiological acute response to exercise rather than pathological sign [2,3,4]. Although the mechanisms underlying the release of cTn following exercise in apparently healthy individuals are not completely understood, previous research suggested potential mechanisms, among them: changes in membrane permeability allowing unbound cTn from cytosol to diffuse outside the cells, normal turnover of myocardial cells, cTn degradation producing cellular release, membranous blebs, myocyte apoptosis/necrosis resulting in genuine cardiac injury or cross-reaction with skeletal troponin [3,5].

Post-exercise elevations of cTn have been noted also in adolescent athletes [6]. However, differences between adolescents and adults are inconsistent [4,6,7,8]. In this regard, it has been previously suggested that higher exercise-induced elevations of cTn in the younger might be attributable to the immaturity of adolescents myocardium, since it would experience greater stress in response to an increased myocardial workload compared with the adults [9]. Based on this hypothesis, it is possible that younger athletes respond to exercise with higher elevations of this biomarker than adults. Notwithstanding that, prior literature has also linked exercise-induced elevations of cTn above the upper reference limit to higher mortality and cardiovascular events in older athletes [10]. In this case, higher elevations in the older might be related to underlying, subclinical, cardiac pathology [11].

Understanding how, when, and why cTn elevates after exercise is relevant for the triage of athletes who develop chest pain that mimics cardiac injury after exercise, and who might have serum cTn drawn in the emergency departments (EDs). Furthermore, a better knowledge of the relationship between exercise-induced elevations of cTn and participants’ age might contribute to a better understanding of the phenomenon and its mechanisms. For these reasons, the purpose of this study was to compare the release of cTnT after a distance-trial test of 45 min swimming between two cohorts of adolescent and adult swimmers. Based on previous studies, our hypothesis was that adolescents would respond to exercise with higher peak elevations of cTn, supporting the theory that the immature myocardium experiences higher workload compared with the adults.

## 2. Materials and Methods 

### 2.1. Participants

A convenience sample of thirty-two trained male swimmers were recruited for this study. All participants and parents of those under the age of 18 provided their informed consent. All swimmers trained in the same club and competed at the regional level. Participants were divided into adolescent (<18 years) and adult (≥18 years) groups (see participant characteristics in Table 1). The study was approved by the Ethical Committee of Clinical Research of Sports Administration of Catalonia (02/2018/CEICGC).

### 2.2. Procedures

Before the intervention, participants underwent anthropometric assessment and a resting 12-lead electrocardiogram (Click ECG BT 12 channel, Milano, Italy). Swimmers performed a self-paced 5 min swimming warm-up (<60% of %HRmax) [6], followed by a distance-trial test of 45 min continuous swimming, and venous blood samples were drawn before, immediately and at 3 h after exercise. Participants were asked to avoid vigorous exercise during the 48 h prior to the intervention. Serum cTnT was determined using a Troponin T hs STAT immunoassay in a Cobas E 601 analyzer (Roche Diagnostics, Penzberg, Germany, range 3–10,000 ng/L). The upper reference limit for cTnT was 13.5 ng/L [11]. Concentrations below the limit of detection were set to 1.5 ng/L for statistical analyses. Heart rate during the test was recorded using Polar OH1™ optical heart rate sensors (Polar Electro Oy, Kempele, Finland). Maximum heart rate for relative intensities was calculated using Tanaka’s formula of 208 − (0.7 × Age). A year after the intervention, participants were interviewed to identify cases of cardiac signs or symptoms.

### 2.3. Statistical Analysis

Analyses were performed using R version 3.5.1 (R Foundation for Statistical Computing, Vienna, Austria). Data were visually inspected to detect abnormal values, and Shapiro-Wilk test was used to assess normality. Accordingly, data were presented as mean ± SD [range] or median (interquartile range) [range], as appropriate. Participant characteristics and exercise load were compared among groups using *t*-test for independent samples. Then, time differences within each group were compared using non-parametric Friedman test for repeated measures and pairwise comparisons between moments were made with Wilcoxon signed rank tests applying Bonferroni corrections. Differences between groups in absolute cTn and its changes (Δ 0 h and Δ 3 h) were analyzed using Kruskal-Wallis rank sum test. Associations between cTn elevations and the rest of the variables were assessed using Spearman correlation coefficients (ρ). Statistical significance in all comparisons was assumed when *p* < 0.05.

## 3. Results

Participant characteristics and exercise load during the distance trial are summarized in Table 1. There were group differences in age, body mass, body mass index and maximal heart rate, and training experience was comparable between groups. During the distance trial, adolescents covered more distance and achieved higher cardiac intensity than adults, though the rating of perceived exertion was comparable between groups.

**Figure 1:**
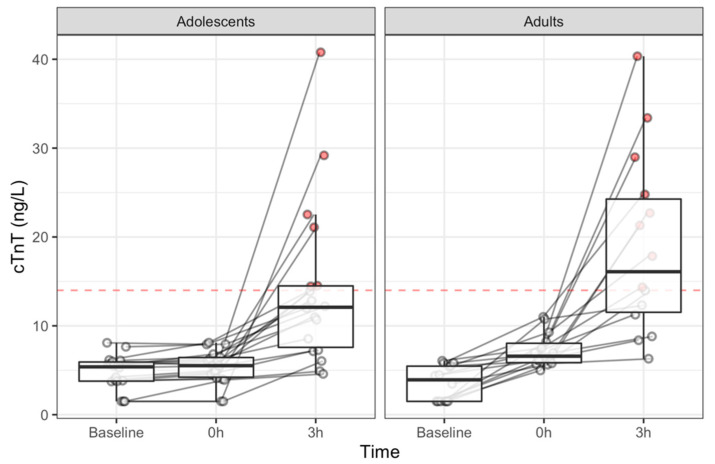
Individual points of cTnT by group. Red horizontal line and red dots denote the cutoff value for myocardial infarction (MI) of 13.5 ng/L and above values, respectively.

Concentrations of cTnT changed significantly over time (*χ*^2^ = 59.5, *p* < 0.001). Concretely, baseline concentrations were comparable between adolescents and adults (*χ*^2^ = 2.2; *p* = 0.13), and in both groups it raised immediately after and at 3 h post exercise. Furthermore, immediate changes (Δ0 h) were different between groups (*χ*^2^ = 18.1; *p* < 0.001), slightly higher in the adults (Figure 1). Peak changes (Δ3 h), however, were comparable between groups (*χ*^2^ = 3.2; *p* = 0.074). Peak cTnT concentrations were observed at 3 h post-exercise in all participants, and 14 (44%) subjects exceeded the cutoff value for myocardial infarction (MI) (6 (33%) adolescents, 8 (57%) adults). Peak changes (Δ3 h) were uncorrelated with age (ρ = 0.19, *p* = 0.29), training experience (ρ = −0.21, *p* = 0.24), body mass index (ρ = 0.05, *p* = 0.8), distance (ρ = −0.1, *p* = 0.59), peak heart rate (ρ = 0.06, *p* = 0.74), and mean heart rate (ρ = 0.06, *p* = 0.73).

## 4. Discussion

The purpose of this study was to compare the exercise-induced release of cTnT between adolescents and adults. Our main finding was that, although having comparable training experience and baseline values of cTnT, adults exercised at lower absolute and relative intensity, but presented higher immediate concentrations of cTnT after exercise than adolescents. However, peak concentrations were observed at 3 h post exercise in all participants and were comparable between groups.

Reference values of cTn seem to be lower in the young, and this might be explained by a positive association between age and the prevalence of cardiovascular diseases [4,11]. However, our results did not support those previous statements since swimmers had comparable basal concentrations of cTnT. Even though this study was limited by a small sample size, it is not the first research finding no age-differences in apparently healthy, trained, young participants [7]. Interestingly though, although peak cTnT was found in all participants at 3 h post exercise, we detected higher elevations immediately after exercise in the adults. Since peak elevations were comparable among groups, this suggests that serological cTnT elevations might appear earlier after exercise in adults compared with adolescents. 

It has been suggested that higher concentrations of post-exercise cTn in the older may represent myocardial injury because of underlying, subclinical, cardiac pathology [11]. However, all participants in this study had normal resting ECG at the beginning of the study. Furthermore, one year after the intervention, none of them reported to have had cardiac symptoms or events during this period. Assuming that our participants were healthy, these evidences align with the theory of transient elevations of cTnT after exercise being a physiological acute response to exercise rather than a sign of myocardial injury [3]. However, this study did not include other examinations of myocardial health such as echocardiographies or other biomarker assessments such as N-terminal prohormone of brain natriuretic peptide (NT-proBNP), creatine kinase myocardial band (CK-MB), or c-reactive protein (CRP). Due to this limitation, we cannot be certain to discard a possible role of underlying, undetected scenarios such as cardiac hypertrophy and thyroid dysfunction [12].

In the present study, cTnT raised in all participants after exercise, and peak elevations at 3 h post exercise were comparable among groups. This is contrary to our expectations, since previous authors suggested that the less mature myocardium in the younger might be more susceptible to exercise-induced elevations of cTn [8,9]. Interestingly though, a recent study reported higher exercise-induced elevations of cTnT in late puberty, suggesting that this might be explained by the higher relative intensities during exercise achieved in this group [13]. In line with this study, our adolescents group also achieved higher cardiac intensities during the test. In spite of that, we found no association between exercise intensity and peak elevations of cTnT. This finding seems interesting, since we expected post-exercise cTnT concentrations to be directly associated with exercise intensity, as has been reported in previous studies [4]. Thus, our results not only suggest that adults elevate cTn before adolescents, but also that the association between cTnT elevations and exercise intensity might not depend on age. 

Almost one-half of the participants in this study (44%) exceeded the upper reference limit for cTnT in the third blood extraction. Previous studies involving similar assessments and exercise exposures also found high rates of positive detection in both young and adult trained participants [4,6,7,8,13]. This is particularly relevant for the triage of athletes who develop chest pain that mimics cardiac injury after exercise, and who might have serum cTn drawn in the EDs. For these reasons, future studies should address the limitations present in this research, including a more exhaustive clinical screening of the participants in order to discard or identify a potential role of underlying pathology.

Finally, in the authors’ opinion, the main strength of this study is that that we could compare the elevations of cTnT in a cohort of trained swimmers that allowed comparisons between age groups, and this made it possible for us to identify an earlier elevation in the adults compared with the adolescents. The main limitations in this study, by contrast, have been mentioned in the above paragraphs. We could not perform an exhaustive cardiac screening including echocardiography, additional biochemical analyses, or assessments of maturational status, as has been done or mentioned in some previous studies [6,12,13]. Additionally, we did not perform cTnT measurements beyond the 3 h post exercise. Consequently, the limited sampling points in our design imply a potential under-estimation error in the peak cTnT concentrations, as has been previously suggested by others [14,15].

## 5. Conclusions

In conclusion, in this study we observed age differences in the immediate elevation of cTnT after exercise, but not in its peak elevations, at 3 h post exercise. Although participants were apparently healthy based on resting ECG and a 1 year term follow-up, future works might continue this line of research and explore the association between immediate elevations of cTnT, age, and health.

## Figures and Tables

**Table 1:** Summary of participants’ characteristics and exercise load.

	Adolescents (*n* = 18)	Adults (*n* = 14)	Between-Groups
Participant Characteristics			
Age (years)	14 ± 3 [11–17]	35 ± 9 [23–52]	*p <* 0.001
Training experience (years)	7 ± 2 [2–11]	6 ± 2 [4,5,6,7,8,9]	*p* = 0.78
Body height (cm)	172.3 ± 9.6 [151–187]	177.4 ± 5.5 [169–187]	*p* = 0.065
Body mass (kg)	60.6 ± 10.2 [44.5–77.8]	71 ± 4.4 [66–81]	*p <* 0.001
Body mass index	20.3 ± 2.2 [16.9–24.3]	26.6 ± 1.3 [20–24.8]	*p* = 0.001
Exercise Load			
Distance (m)	1862 ± 276 [1250–2300]	1650 ± 239 [1320–2060]	*p* = 0.027
Mean relative heart rate (% HRmax)	89 ± 5 [75–99]	82 ± 4 [75–88]	*p* < 0.001
Peak relative heart rate (% HRmax)	95 ± 6 [82–110]	91 ± 4 [82–96]	*p* = 0.037
Rating of perceived exertion	7 ± 2 [3–10]	8 ± 1 [6–10]	*p* = 0.24
Cardiac Troponin			
Basal cTnT (ng/L)	5.39 (2.15) [1.5–8.09]	3.93 (3.97) [1.5–6.07]	*p* = 0.13
Δ 0 h post-exercise cTnT (ng/L)	0.38 (0.62) [−0.46–2.41]	3.69 (2.78) [0.57–9.25)	*p* < 0.001
Δ 3 h post-exercise cTnT (ng/L)	5.08 (6.37) [1.07–34.67)	11.64 (13.62) [2.83–38.84]	*p* = 0.074

Participant characteristics and exercise load data are described as mean ± standard deviation [range], whereas cTn data are described as median (interquartile range) [range]. Δ 0 h post-exercise cTnT, absolute change from baseline to immediately after exercise; Δ 3 h post-exercise cTnT, absolute change from baseline to 3 h post-exercise.

## Data Availability

The data presented in this study are available on request to the authors. Some variables are restricted to preserve the anonymity of study participants.
